# Serum testosterone level correlates with left ventricular hypertrophy in older women

**DOI:** 10.3389/fendo.2022.1079043

**Published:** 2023-01-06

**Authors:** Iwona Szadkowska, Agnieszka Guligowska, Anna Jegier, Marek Pawlikowski, Hanna Pisarek, Katarzyna Winczyk, Tomasz Kostka

**Affiliations:** ^1^ Department of Sports Medicine, Medical University of Lodz, Lodz, Poland; ^2^ Department of Geriatrics, Healthy Ageing Research Centre, Medical University of Lodz, Lodz, Poland; ^3^ Department of Immunoendocrinology, Medical University of Lodz, Lodz, Poland; ^4^ Department of Neuroendocrinology, Chair of Laboratory and Molecular Diagnostics, Medical University of Lodz, Lodz, Poland

**Keywords:** sex hormones, gonadotropins, cardiac remodeling, echocardiogram, aging

## Abstract

**Introduction:**

Sex hormones may play an important role in age-related cardiac remodeling. However, their impact on cardiac structure and function in females of advanced age still remains unclear. The aim of this study is to evaluate the relationship between sex hormones level and echocardiographic parameters in older women with concomitant cardiovascular diseases.

**Materials and Methods:**

The study group included 52 community-dwelling women with mean age 79.5 ± 2.8 years, consecutive patients of an outpatient geriatric clinic. In all the subjects, a transthoracic echocardiogram was performed and serum testosterone, estradiol, follicle-stimulating hormone, luteinising hormone, dehydroepiandrosterone sulphate, and cortisol levels were determined.

**Results:**

Testosterone level correlated positively with interventricular septum diastolic dimension (IVSd) (rS=0.293, p<0.05), left ventricular mass index (rS=0.285, p<0.05), E/E’ ratio (rS=0.301, p<0.05), and negatively with E’ (rS=-0.301, p<0.05). Estradiol level showed a positive correlation with the posterior wall dimension (rS=0.28, p<0.05). Besides, no significant correlations between clinical or echocardiographic parameters and other hormones were observed. Female subjects with diagnosed left ventricular hypertrophy (LVH) (n=34) were characterized by a significantly higher rate of hypertension (p=0.011), higher waist-to-height ratio (p=0.009), higher testosterone level (0.82 vs. 0.48 nmol/L, p=0.024), higher testosterone/estradiol ratio (16.4 vs. 9.9, p=0.021), and received more anti-hypertensive drugs (p=0.030). In a multiple stepwise logistic regression, the best determinants of LVH were the presence of hypertension (OR=6.51; 95% CI 1.62-26.1), and testosterone level (OR= 6.6; 95% CI 1.19-36.6).

**Conclusions:**

Higher serum testosterone levels may contribute to pathological cardiac remodeling, especially in hypertensive women. Estradiol, gonadotropins, DHEAS, and cortisol were not related to echocardiographic parameters.

## 1 Introduction

Cardiovascular diseases are consistently the leading cause of death in developed countries (1). At the same time, their incidence rises rapidly in people of advanced age. Chronic diseases such as hypertension (HT), coronary artery disease (CAD), or heart failure (HF) affect most of the elderly population (1–3).

Heart failure is currently the major health problem affecting populations worldwide in terms of high economic costs as well as poor prognosis and reduced quality of life. It is estimated that the prevalence of HF is about 1% for people aged < 55 years and rises with age to above 10% in the elderly aged > 70 years. Additionally, more than one-half of HF patients are females (2). The predominant causes of HF are HT and CAD, with a higher prevalence of HF with reduced ejection fraction (HFrEF) in men because of underlying ischemic processes, and HF with preserved ejection fraction (HFpEF) in women, mostly due to hypertension (2–4). Chronic pressure overload in hypertensive patients leads to left ventricular hypertrophy (LVH) with its implications such as impaired left ventricular (LV) diastolic function and the development of clinical symptoms of HF (3). The presence of LVH is considered as one of the indicators of hypertension-mediated organ damage and an important risk factor for cardiac death in the general population, and the regression of LVH improves patients’ prognosis (3, 5).

Sex steroid hormones play an important role in regulating the function of the whole human body, including the heart and vessels. The protective role of estrogens results in lower cardiovascular risk in premenopausal women than in men at the same age, however, this relationship changes dramatically after menopause (6). In females, the association between testosterone and risk or course of cardiovascular diseases has not yet been precisely established, especially in advanced age. Significantly more studies regarding the physiological role of testosterone focused on men, where generally beneficial effects have been demonstrated in the context of increasing skeletal muscle mass and strength, reducing body fat percentage, improving cardiac contractility and relaxation of the heart and vessels (7–10). On the other hand, the use of anabolic-androgenic steroids, as in some sports, while increasing muscle mass and performance, has a number of well-known adverse cardiovascular effects (11, 12).

However, the results of the studies concerning the relationships between endogenous or exogenous testosterone in therapeutic doses and cardiovascular risk are still ambiguous (7, 8, 13–16). Recently, particular attention has been focused on HF patients. In this group, testosterone supplementation is considered as an additional way of pharmacological treatment to improve cardiac health, but the results are still inconclusive, even with reported possible deterioration of heart function (17–20).

Likewise, even less is known on the potential relationship of gonadotropins to cardiovascular risk and cardiac function in very old age (21–23). Therefore, the aim of our study was to assess the relationship between serum levels of sex hormones and echocardiographic parameters of the cardiac structure and function in women of advanced age, in relation to concomitant cardiovascular diseases.

## 2 Materials and methods

### 2.1 Study population

A group of 52 community-dwelling women of advanced age (mean age 79.5 ± 2.8; range 76-87 years), consecutive patients of an outpatient geriatric clinic were recruited for the study. The inclusion criteria defined were the following: female sex, age over 75 years, stable clinical status, no history of hormonal treatment.

The exclusion criteria were: mental or functional inability to participate in the study, a history of panhysterectomy, diagnosis of hypo- and hyperthyroidism, adrenal or pituitary disorders, hemodialysis therapy, hormone therapy (including use of anabolic-androgenic steroids) and lack of patient’s consent. To obtain an adequate echocardiographic measurement and eliminate factors potentially affecting cardiac parameters, patients with atrial fibrillation or severe arrhythmias were excluded from the study.

Laboratory tests were performed on fasting serum samples (within one hour after blood collection). Total cholesterol (TC), high-density lipoprotein cholesterol (HDL-C), low-density lipoprotein cholesterol (LDL-C), triglycerides (TG), and glucose (GLU) were measured using the DXC700 analyser, Beckman Coulter, Brea, USA. All the tests and analysers applied in the study were certified for routine *in-vitro* diagnostic.

Body mass index (BMI) and waist to height ratio (WHtR) were calculated using the collected anthropometric data (body mass and height were measured using RADWAG personal weight scales (WPT60 150OW; Radwag Balances and Scales, Radom, Poland) and waist circumference was assessed using the SECA measuring tape (SECA Deutschland, Hamburg, Germany).

A transthoracic echocardiogram was performed and serum hormones levels were determined in all the subjects who met the criteria of the study. Elevated blood pressure (BP) was defined according to the European guidelines for arterial hypertension: ≥ 140 mmHg for systolic blood pressure (SBP) and/or ≥ 90 mmHg for diastolic blood pressure (DBP) (3).

The study protocol was approved by the Bioethics Committee of the Medical University of Lodz in accordance with the Declaration of Helsinki (RNN/345/08/KB) and all the patients gave their written consent for participation in this study.

### 2.2 Determination of hormone levels

Serum levels of estradiol, testosterone, follicle-stimulating hormone(FSH), luteinising hormone (LH), dehydroepiandrosterone sulphate (DHEAS), and cortisol were determined in blood samples taken in the morning from the cubital vein from overnight fasting patients. Hormone concentrations were determined using the technology based on competitive or sandwich chemiluminescence immunoassays (CLIA). Measurements were made with sets from Saluggia (Italy or Stillwater, MN, USA) on the LIAISON XL analyzer from DiaSorin Inc.). The testosterone to estradiol ratio (T/E) was calculated by dividing the respective total serum levels after converting both to pmol/L.

### 2.3 Echocardiographic assessment

Transthoracic echocardiography was performed in all the patients by the same experienced cardiologist with the use of the Vivid S70 ultrasound system (GE Medical Systems, 2018). The parameters of cardiac structure and function were assessed according to the current recommendations (24, 25). The following internal cardiac dimensions were obtained with the use of two-dimensional linear measurements: interventricular septal diastolic dimension (IVSd), left ventricular diastolic dimension (LVDd), posterior wall diastolic dimension (PWd), and left atrial (LA) antero-posterior diameter. Relative wall thickness (RWT) was calculated according to the following formula: 2 × PWd divided by LVDd. Left ventricular mass was automatically calculated using the Devereux formula and indexed to body surface area (LV mass index – LVMI). Left ventricular hypertrophy (LVH) was defined according to the criteria as LVMI > 95 g/m^2^ for women (24). Left ventricular end-diastolic volume (LVEDV), LV end-systolic volume (LVESV), LV ejection fraction (LVEF) were determined using the modified biplane Simpson’s method from the apical four- and two-chamber views. LVEDV and LVESV were indexed to body surface area - LVEDV index (LVEDVI) and LVESV index (LVESVI). Left atrial volume was measured using the biplane area-length method and divided by body surface area (LA volume index – LAVI). The peak early (E), and late-atrial (A) diastolic velocities were obtained by pulsed-wave Doppler from the mitral inflow profile. The E/A ratio was calculated. Pulsed tissue Doppler imaging (TDI) was used to measure the LV peak systolic velocity (S’) and early diastolic velocity (E’) from the median (septal) and lateral mitral annular velocities. Then, the mean values of these parameters were calculated. Additionally, the average E/E’ ratio from septal and lateral measurements was calculated. Systolic and diastolic LV function was interpreted in accordance with the current guidelines (2, 24, 25).

### 2.4 Statistical analysis

The total sample size required to determine whether a correlation coefficient differs from zero (α (two-tailed) =0.05; β=0.20; r=0.4) is 47. The normality of distribution was verified using the Shapiro-Wilk test. Not normally distributed continuous variables were presented by median and the first (25%) to the third (75%) quartile. Spearman correlations between hormones level and numerical data were calculated. The quantitative variables (between the hypertrophy groups), were compared using the Mann-Whitney U-test. The occurrence of differences between the groups was assessed using a one-way ANOVA, the chi-square test, or Fischer’s Exact test.

After log transformation of the age and testosterone level, multiple linear regression was performed to assess what independent variables determine IVSd, E’, E/E’ ratio and LVMI.

Stepwise logistic regression (odds ratios and corresponding 95% confidence intervals (95%CI) was used to assess which independent variables predicted the presence of hypertrophy. When building the model, the following variables were taken into account: age, testosterone level and hypertension.

Statistical significance was set at p ≤ 0.05. The analyses were performed using Statistica 13.1 (StatSoft Polska, Cracow, Poland)

## 3 Results

The baseline characteristics of the study group are presented in **Table 1**. All the subjects (n=52) were females of advanced age (median 79 years), with median BMI value of 28.7 kg/m^2^, SBP 136 mmHg and DBP 74.5 mmHg. HT was diagnosed in 67% of the participants, and the most commonly used anti-hypertensive drug groups were angiotensin-converting enzyme inhibitors (ACEI) or angiotensin receptor blockers (ARB) and beta-blockers.

**Table 1:** Baseline characteristics of the study group and divided into a group with and without LV hypertrophy.

Variables	All n=52	with LV hypertrophy; n=34	without LV hypertrophy; n=18	p
Age [years]	79 (77-81)	79 (77-81)	79 (77-80)	ns
BMI [kg/m^2^]	28.7 (24.6-31.2)	29.8 (26.3-32.0)	25.7 (23.5-30.4)	ns
WHtR	0.57 (0.55-0.63)	0.60 (0.56-0.64)	0.55 (0.54-0.57)	0.009
SBP [mmHg]	136 (121-149)	136 (124-150)	136 (118-148)	ns
DBP [mmHg]	74.5 (70-82.5)	75 (70-83)	72 (67-77)	ns
SBP ≥ 140 mmHg; n (%)	27 (51.9)	19 (55.8)	8 (44.4)	ns
DBP ≥ 90mmHg, n (%)	3 (5.8)	2 (5.9)	1 (5.5)	ns
Cholesterol [mg/dL]	200 (177 – 244)	193 (177 – 224)	211.5 (195 - 252)	ns
HDL [mg/dL]	65.5 (58 - 73.5)	64.5 (56 – 72)	66.5 (61 - 79)	ns
LDL [mg/dL]	111 (87 – 147)	105.5 (84 – 134)	121 (107 – 162)	ns
TG [mg/dL]	99.5 (81.5 - 141.5)	101.5 (80 - 151)	99.5 (94 121)	ns
Glucose [mg/dL]	101 (91 – 108)	102 (93 - 116)	97.5 (91 – 106)	ns
Hypertension; n (%)	35 (67.3)	27 (79.4)	8 (44.4)	0.011
Diabetes; n (%)	10 (19.2)	7 (20.6)	3 (16.7)	ns
Coronary artery disease; n (%)	5 (9.6)	4 (11.7)	1 (5.5)	ns
Medications; n (%): ACEI/ARB	31 (59.6)	23 (67.6)	8 (44.4)	ns
Beta-blockers	31 (59.6)	23 (67.6)	8 (44.4)	ns
CCB	19 (36.5)	16 (47.0)	3 (16.6)	0.030
Diuretics	13 (25)	9 (26.4)	4 (22.2)	ns
MRA	8 (15.4)	6 (17.6)	2 (11.1)	ns
Statins	29 (55.8)	20 (58.8)	9 (50)	ns
Serum hormone levels: estradiol [pmol/L]	52 (44-63.7)	53.7 (48.2 - 65.2)	46.4 (43.3 - 57.3)	ns
testosterone [nmol/L]	0.73 (0.44-0.94)	0.82 (0.49 - 1.06)	0.48 (0.38 - 0.89)	0.024
testosterone/estradiol ratio	12.4 (7.77 – 18.6)	16.4 (10.8 - 19.7)	9.9 (6.26 - 12.4)	0.021
FSH [mIU/mL]	89.3 (70.5-105)	89.3 (76.5 - 107)	88.4 (67.6 - 98.5)	ns
LH [mIU/mL]	19 (16.1-26.1)	19.7 (16.3 - 26.8)	17.8 (15.0 - 23.2)	ns
DHEAS [µg/dL]	34 (22.5–57.9)	36.4 (26.1 - 60.3)	25.6 (15.6 - 54.5)	ns
Cortisol [µg/dL]	13.8 (9.33–17.8)	14.3 (9.48 - 21.5)	12.5 (7.27 - 15.6)	ns

The quantitative values are expressed by median and interquartile difference, and qualitative values as number and percentage.

BMI, body mass index; WHtR, waist-to-height ratio; SBP, systolic blood pressure; DBP, diastolic blood pressure; ACEI, angiotensin-converting enzyme inhibitors; ARB, angiotensin receptor blockers; CCB, calcium channel blockers; MRA, mineralocorticoid receptor antagonist; FSH, follicle-stimulating hormone; LH, luteinising hormone; DHEAS, dehydroepiandrosterone sulphate; LDL, low-density lipoprotein; HDL, high-density lipoprotein.

Elevated BP levels occurred in about one-half of the patients with reference to SBP and in ca. 6% to DBP, however, the values only slightly exceeded the targeted cut-off points for the general population. Diabetes was manifested in about 20%, and CAD in about 10% of the study group. The cholesterol and glucose results were within the normal levels or only slightly above the normal range.

The median (interquartile difference 25%-75%) for hormone levels was as follows: estradiol 52 (44-63.7) pmol/L, testosterone 0.73 (0.44–0.94) nmol/L, FSH 89.3 (70.5-105) mIU/mL, LH 19 (16.1-26.1) mIU/mL, DHEAS 34 (22.5–57.9) µg/dL, cortisol 13.8 (9.33–17.8) µg/dL, and for testosterone/estradiol ratio was 12.4 (7.77 – 18.6).

To perform a more precise analysis, the study group was divided according to the presence (n=34) or absence (n=18) of LVH. The women with LV hypertrophy were characterized by a significantly greater incidence of HT, higher WHtR, and received more anti-hypertensive drugs (especially calcium channel blockers) in comparison to the non-LVH subgroup. Regarding the sex hormones, significantly higher serum testosterone level and testosterone/estradiol ratio were observed in LVH patients. There were no notable differences between the subgroups in relation to age, BMI, BP values, the incidence of diabetes or CAD, and other hormones levels. There were no differences in cholesterol, triglycerides and glucose levels (**Table 1**).

**Table 2** shows the results of echocardiographic measurements, which were generally within the normal range. Relatively high values were reached for RWT and LVMI, calculated from wall thickness, LV volumes and additionally body surface area for LVMI; in about 65% of the females, LVH was diagnosed. The subgroup with LVH was characterized by a significantly lower LV dimension (LVDd), higher LV walls thickness (IVSd and PWd), RWT, and LVMI. Additionally, the parameters of LV diastolic function (E’, E/E’, LA) were worse in this subgroup in comparison to the women without LVH.

**Table 2:** Echocardiographic characteristics of the study group and divided into a group with and without LV hypertrophy.

Variables	All n=52	with LV hypertrophy; n=34	without LV hypertrophy; n=18	p
IVSd [cm]	1.1 (1.0 - 1.1)	1.1 (1.1 - 1.2)	1.0 (0.9 - 1.0)	0.0000
LVDd [cm]	4.6 (4.4 - 5.0)	4.7 (4.5 - 5.0)	4.45 (4.30 - 4.70)	0.0328
PWd [cm]	1.0 (0.9 – 1.0)	1.0 (1.0 - 1.1)	0.9 (0.90 - 1)	0.0004
RWT	0.44 (0.41-0.48)	0.46 (0.42 - 0.49)	0.42 (0.39 - 0.44)	0.0044
LA [cm]	3.60 (3.2-3.9)	3.70 (3.5 - 4)	3.2 (3.1 - 3.6)	0.0014
LAVI [ml/m2]	26.2 (20.2 -34.6)	27.7 (22.1 - 35.1)	24 (19.1 - 26.6)	ns
LVEDV [ml]	64 (51-78)	64 (49 - 77)	62.5 (51 - 78)	ns
LVEDVI [ml/m2]	37.1 (30.7-44.6)	37.6 (30.7 - 42.1)	36.2 (30.7 - 46.4)	ns
LVESV [ml]	24.0 (19-30)	24 (18 - 32)	24 (19 - 29)	ns
LVESVI [ml/m2]	13.8 (12-17.3)	14.9 (11.9 - 16.5)	13.8 (12.7 - 17.4)	ns
LVEF [%]	61 (58 -63)	60.5 (58 - 63)	61 (60 - 64)	ns
LVMI [g/m2]	114 (102-123)	118 (109 - 132)	94 (84 - 114)	0.0000
E [m/s]	0.59 (0.49-0.71)	0.58 (0.49 - 0.73)	0.61 (0.51 - 0.65)	ns
E/A ratio	0.69 (0.59-0.81)	0.68 (0.57 - 0.8)	0.73 (0.63 - 0.82)	ns
S’ [m/s]	0.08 (0.08-0.09)	0.08 (0.08-0.09)	0.08 (0.07 - 0.1)	ns
E’ [m/s]	0.07 (0.06-0.08)	0.06 (0.06 - 0.08)	0.08 (0.07 - 0.09)	0.0067
E/E’ ratio	8.64 (7.16-10.3)	9.19 (8.16 - 10.5)	7.09 (6.76 - 8.99)	0.0013
LV hypertrophy; n (%)	34 (65.4)	34 (100)	0	0.0000

The quantitative values are expressed by median and interquartile difference and qualitative values as number and percentage.

IVSd, interventricular septal diastolic dimension; LVDd, left ventricular diastolic dimension; PWd, posterior wall diastolic dimension; RWT, relative wall thickness; LA, left atrial anteroposterior diameter; LAVI, left atrial volume index; LVEDV, left ventricular end-diastolic volume; LVEDVI, left ventricular end-diastolic volume index; LVESV, left ventricular end-systolic volume; LVESVI, left ventricular end-systolic volume index; LVEF, left ventricular ejection fraction; LVMI, left ventricular mass index; E, early mitral diastolic inflow velocity; E/A ratio, ratio of early to late mitral inflow velocities; S’, average systolic mitral annular velocity; E’, average early diastolic mitral annular velocity; E/E’ ratio, an average ratio of early mitral diastolic inflow velocity to early diastolic mitral annular velocity; LV, left ventricle.

**Figure 1:**
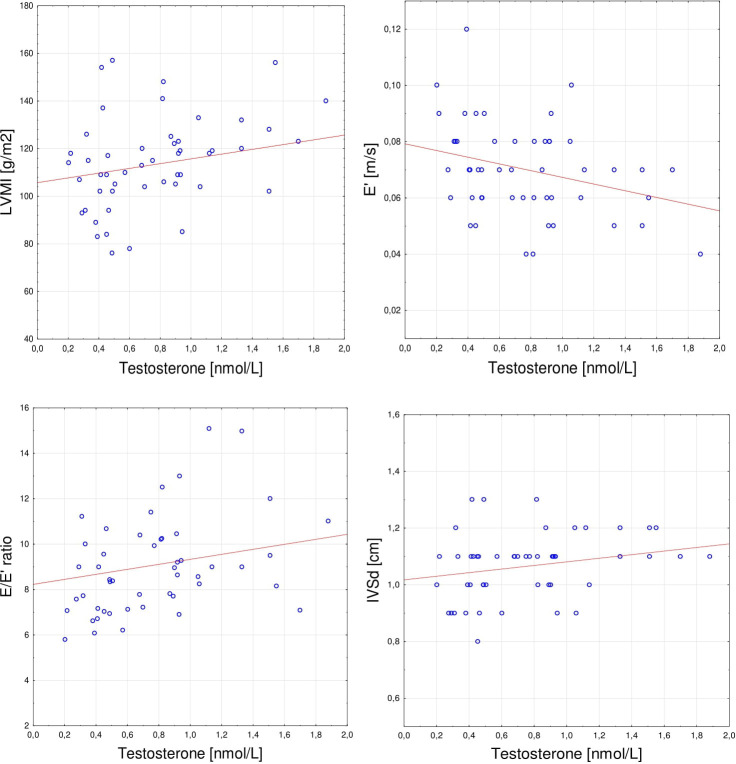
Spearman’s correlations between serum testosterone level and **(A)** LVMI (r= 0.285, p < 0.05), **(B)** E’(r= -0.301, p < 0.05); **(C)** E/E’ ratio (r= 0.301, p < 0.05); **(D)** IVSd (r= 0.293, p < 0.05). LVMI, left ventricular mass index; E’, average early diastolic mitral annular velocity; E/E’ ratio, average ratio of early mitral diastolic inflow velocity to early diastolic mitral annular velocity; IVSd, interventricular septal diastolic dimension.

The correlations of sex hormones levels with echocardiographic parameters and clinical features are presented in **Table 3**. Estradiol level was significantly positively correlated with PWd (rS= 0.280, p < 0.05). Positive correlations were found between testosterone level and IVSd (rS= 0.293, p < 0.05), LVMI (rS= 0.285, p < 0.05), E/E’ ratio (rS= 0.301, p < 0.05), and negative with E’ (rS= -0.301, p < 0.05). The testosterone/estradiol ratio correlated positively with E/E’ ratio (rS = 0.314, p < 0.05) and inversely with E’ (rS= -0.333, p < 0.05). Additionally, there were no significant associations between echocardiographic parameters and other hormones levels – FSH, LH, DHEAS, and cortisol. The correlations observed for testosterone are presented in detail in **Figure 1**.

**Table 3:** Spearman’s correlations of serum hormones levels with echocardiographic parameters and clinical features.

Variables	Estradiol [pmol/L]	Testosterone [nmol/L]	Testosterone/estradiol ratio
IVSd [cm]	0.275	0.293*	-0.270
LVDd [cm]	0.101	0.106	-0.092
PWd [cm]	0.280*	0.176	-0.095
RWT	0.190	0.172	0.143
LA [cm]	0.097	0.183	0.097
LAVI [ml/m2]	0.071	0.115	0.180
LVEDV [ml]	0.020	-0.121	-0.086
LVEDVI [ml/m2]	-0.021	-0.142	-0.106
LVESV [ml]	0.096	-0.054	-0.051
LVESVI [ml/m2]	0.067	-0.065	-0.053
LVEF [%]	-0.115	-0.117	-0.106
LVMI [g/m2]	0.240	0.285*	0.254
E [m/s]	-0.134	-0.008	-0.030
E/A ratio	-0.032	-0.086	-0.179
S’ [m/s]	-0.110	-0.145	-0.073
E’ [m/s]	-0.154	-0.301*	-0.333*
E/E’ ratio	0.087	0.301*	0.314*
Age [years]	0.069	0.068	0.108
BMI [kg/m^2^]	0.179	0.076	0.039
WHtR	0.212	0.120	0.106

* p < 0.05; IVSd, interventricular septal diastolic dimension; LVDd, left ventricular diastolic dimension; PWd, posterior wall diastolic dimension; RWT, relative wall thickness; LA, left atrial anteroposterior diameter; LAVI, left atrial volume index; LVEDV, left ventricular end-diastolic volume; LVEDVI, left ventricular end-diastolic volume index; LVESV, left ventricular end-systolic volume; LVESVI, left ventricular end-systolic volume index; LVEF, left ventricular ejection fraction; LVMI, left ventricular mass index; E, early mitral diastolic inflow velocity; E/A ratio, ratio of early to late mitral inflow velocities; S’, average systolic mitral annular velocity; E’, average early diastolic mitral annular velocity; E/E’ ratio, average ratio of early mitral diastolic inflow velocity to early diastolic mitral annular velocity; BMI, body mass index; WHtR, waist-to-height ratio.

In multiple linear regression, testosterone significantly determined IVSd (β=0.29; p=0.039), E’ (β= -0.35; p=0.01), E/E’ ratio (β=0.3; p=0.036), while hypertension and age did not enter the model. However, none of the variables entered the model describing LVMI, and the variables remained statistically insignificant.

In a stepwise logistic regression, a higher testosterone level (OR=6.6; 95%CI: 1.19-36.6; p= 0.031) and hypertension (OR=6.51; 95%CI: 1.62-26.1; p=0.008) were associated with LVH occurrence.

## 4 Discussion

Sex hormones may play a crucial role in gender-specific cardiac aging. It is worth emphasizing that the age-related remodeling of the heart muscle in the form of hypertrophy, increased LV filling pressure, relaxation disorders resulting in diastolic dysfunction, and finally HFpEF are more pronounced in women than in men (26–29). However, the clinical effects of endogenous sex hormones on the cardiac structure and function with respect to concomitant diseases are not clarified, especially in elderly women. In our study, we determined the relationship between sex hormones against the background of other hormones (FSH, LH, DHEAS, cortisol) with echocardiographic indicators of LV hypertrophy, as well as LV systolic and diastolic function in women of advanced age. No systematic association of gonadotropins, estrogen, DHEAS and cortisol to echocardiographic data was observed. In contrast, testosterone was clearly related to echocardiographic parameters that are characteristic for pathological cardiac remodeling.

### 4.1 Estradiol

Numerous studies have revealed the beneficial effect of estrogens or estrogen receptors stimulation on proper cardiovascular function in women. Earlier reports are also confirmed by more recent observations, where the decline of estrogens level and a higher testosterone/estradiol ratio were recognized as the main cause of increased cardiovascular risk in post-menopausal women (30, 31). As an example, in a study conducted by Zhao et al. among 2,834 women at a mean age of 64.9 ± 8 years, a higher endogenous estradiol level was associated with a lower risk of CAD and HF incidence during 12 years of follow-up (31). In relation to the heart muscle, estrogens *via* receptors ERα, ERβ and GPER have an inhibitory effect on cardiac hypertrophy, inflammation, fibrosis, and oxidative stress, which has been confirmed in animal and humans models (32–35). However, as our results show, these relationships are not obvious in very old women in whom age- and disease-related cardiac remodeling may overlap, especially that associated with HT. In our group of females at mean age of 79.5 years, a positive correlation between serum estradiol level and posterior wall thickness was observed, with no association found with other echocardiographic parameters. Moreover, estradiol levels were slightly higher in the subgroup with LVH (p=0.051); however, they did not correlate directly with any of the echocardiographic indicators of potential impairment of LV diastolic function (E, E/A ratio, E’, E/E’ ratio or LA enlargement). The relationships found to some extent differ from most of previous reports. Nevertheless, what has been recently emphasized, the interaction of estradiol with various receptors can result in different effects depending on multiple factors (36). In our study, about 80% of the women with LVH were hypertensive, which is an important fact in the context of the study by Wittnich et al. (37). In this animal study, a weaker protective effect of estradiol on LVH and LV function was observed in the case of HT, which resulted from a 35% lower content of ERβ in myocardial cells. However, these relationships require more detailed analyses, and the impact of estradiol on the heart should be definitely considered in the context of other factors among which testosterone seems to play a significant role.

### 4.2 Testosterone

As it was demonstrated by several studies, endogenous testosterone has a favorable impact on cardiac and vascular function in men, however, in the case of women, the data are inconclusive. Physiologically, in females testosterone production decreases steadily from middle age, and the serum levels are significantly lower than in men, including individuals of advanced age. The results of the reports on the influence of endogenous testosterone on the cardiovascular system in post-menopausal women vary considerably, showing a whole range of effects, from protective to harmful ones (38–40). A recently published epidemiological study has highlighted the direct correlation between concentration of serum total testosterone and all-cause mortality in 93,314 post-menopausal women followed-up for approximately nine years, as opposed to a group of 154,965 men in whom that risk decreased with higher testosterone levels (41). Additionally, in another study including a similar group of women, higher testosterone levels and testosterone/estradiol ratio significantly enhanced the incidence of CAD and HF events over 12 years (31). In the present study, we found a stronger relationship between testosterone and echocardiographic parameters specific for cardiac remodeling in comparison to other hormones. The association was mostly expressed in cardiac hypertrophy, where a positive correlation with IVSd and LVMI was observed. Moreover, the LVH patients were characterized by a higher serum testosterone level, and testosterone/estradiol ratio.

Pressure overload is an essential cause of pathological LVH, and adequate treatment aimed at blood pressure lowering can prevent or even regress it, which decreases the cardiovascular risk (3, 5, 6, 42, 43). We did not show the association between LVH and SBP and DBP. This may be due to received anti-hypertensive treatment. However, as Muiesan et al. concluded, LVH is more frequent in women than in men, regardless of anti-hypertensive treatment and adequate blood pressure control, which indicates the important role of other factors in the pathophysiology of LVH in females (44).

Similarly, the relationship between the indicators of excessive body weight and LVH, particularly expressed in women, is confirmed in the literature (45, 46). It was also observed in our study in the form of higher values of WHtR (p=0.009), as well as a trend towards higher values of BMI (p= 0.058) in the LVH subgroup.

Among the factors analyzed, only the presence of HT and serum testosterone level were found to be independent factors associated with LVH. This may confirm the recently published results of other authors who report a positive correlation between testosterone level and LVMI in a large group of post-menopausal women (47). It is worth noting that the processes dependent on HT and the action of testosterone may overlap, and testosterone itself is also involved in the etiology of HT (48).

Subramanya et al. observed a lower LVEDV and a higher mass/volume ratio assessed in cardiac magnetic resonance imaging, with a higher testosterone level, which indicates concentric LV remodeling resulting from pathological processes (47). In contrast to our study, the participants were younger (mean age 65 ± 9 years), hypertensive in 48% (vs. 67% in our group), had a slightly lower SBD and DBP, a comparable BMI, and higher total testosterone levels (median 0.9 nmol/l). Although the patients’ characteristics and methods differ to some extent, the direction of the observed changes is consistent.

Contrary to men, in females testosterone may negatively influence myocardial relaxation, as was reported by Olszanecka et al. (49). In this study, the free testosterone level was inversely associated with E/A ratio (β= -0.19, p= 0.05), however, it was only one parameter of LV diastolic function obtained. In the previously cited study, similar relationships have been shown, however, only the indirect assessment of cardiac diastolic function was used (LV mass/volume ratio) (47). Our analysis was carried out in compliance with the current guidelines on cardiac imaging using the recommended cardiac measurements, which enabled us to demonstrate a significant negative relationship between testosterone level and E’ and a positive one with E/E’ ratio. The directions of changes in both parameters indicate worsening LV relaxation, which may contribute to diastolic dysfunction and HFpEF. Interestingly, these correlations appeared within the ranges of generally normal values of the above parameters.

### 4.3 Other hormones

In a large prospective cohort study, lower levels of endogenous testosterone and DHEAS in men and DHEAS in postmenopausal women were associated with the development of heart failure (50). After a median of 9.1 years follow-up, higher dehydroepiandrosterone and estradiol levels were associated with increased LVM in older men (47). Likewise, glucocorticoids may play an important role in cardiac remodeling and progression to heart failure (51, 52). The data on potential role of gonadotropins in an aging heart are especially poor. Some recent studies confirm the possibility of direct actions of gonadotropins beyond the reproductive system (53). Elevated serum FSH has been suggested to increase the risk of atherosclerosis and cardiovascular diseases (54, 55). In one available study in young adults, absolute value of circumferential strain assessed on left ventricular short-axis images at the papillary muscle level (mid-ventricular) was positively correlated to FSH (56).

In the present study, we were not able to show any relationship of DHEAS, cortisol, LH and FSH to echocardiographic parameters. This may be due to the fact that previously investigated populations were much younger in comparison to our older women. It cannot be excluded that in the more advanced age the influence of these hormones on the heart may be alleviated (as for DHEAS or cortisol) or clinically negligible. However, the role of hormone receptors in the heart muscle of older subjects needs further detailed studies.

### 4.4 Study strength and limitations

The strength of our research is unique age group of the women examined. Moreover, the study group is quite homogeneous in terms of several characteristics, such as sex, advanced age, and clinical features. A number of cardiac parameters were precisely obtained and analyzed. Thus, it was possible for us to observe subtle relationships that are mostly not found by other researchers.

The limitations of our study include the small sample size. Other shortcomings include a single measurement of hormones level in relation to echocardiographic parameters and cross-sectional character of the study. The strength of the associations found was moderate and may be affected by other factors than those presented in our study. Further studies are required to expand the knowledge on sex hormones affecting the heart muscle in women at an advanced age.

## 5 Conclusions

To sum up, our study conducted among community-dwelling older women as the first in the literature revealed that a higher serum testosterone level may have a negative impact on cardiac structure and function, being associated with LVH and diastolic dysfunction parameters independently of other factors, such as HT or age. The role of estradiol and other hormones in this population have not yet been precisely established. In our study their levels are not related to cardiac function.

Firstly, the results may have important clinical implications in geriatric care, and the measurement of testosterone concentration can be considered in the assessment of individual cardiovascular risk, along with traditional factors, in the population of older women. Secondly, the findings indicate that a multidirectional diagnostics and personalized treatment should be applied especially in individuals with co-existing HT and higher testosterone levels.

## Data availability statement

The raw data supporting the conclusions of this article will be made available by the authors, without undue reservation.

## Ethics statement

The studies involving human participants were reviewed and approved by Bioethical Committee of the Medical University of Lodz, decision number: RNN/363/17/KE. The patients/participants provided their written informed consent to participate in this study.

## Author contributions

Conceptualization, IS, AG, AJ; methodology, AG, IS, MP, KW, TK; investigation, MP, HP, KW, AG, IS; software, AG; validation, IS, AG; formal analysis, AG; data curation, AG, IS; writing—original draft preparation, IS, AG; visualization, AG; supervision, TK, MP, AJ; project administration, TK; funding acquisition, TK, AJ. All authors contributed to the article and approved the submitted version.
